# RAD51 is a potential marker for prognosis and regulates cell proliferation in pancreatic cancer

**DOI:** 10.1186/s12935-019-1077-6

**Published:** 2019-12-27

**Authors:** Xiaomeng Zhang, Ningyi Ma, Weiqiang Yao, Shuo Li, Zhigang Ren

**Affiliations:** 1 Department of Radiation Oncology, Fudan University Shanghai Cancer Center, Shanghai, 200032 China; 2 Department of Oncology, Shanghai Medical College, Fudan University, Shanghai, 200032 China; 3 Department of Interventional Radiology, Zhongshan Hospital, Fudan University, Shanghai, 200032 China; 4 Department of Radiation Oncology, Shanghai Proton and Heavy Ion Center, Shanghai, 201321 China; 5 Shanghai Engineering Research Center of Proton and Heavy Ion Radiation Therapy, Shanghai, 201321 China

**Keywords:** RAD51, KRAS, Aerobic glycolysis, Prognosis, Pancreatic cancer

## Abstract

**Background:**

The DNA damage and repair pathway is considered a promising target for developing strategies against cancer. RAD51, also known as RECA, is a recombinase that performs the critical step in homologous recombination. RAD51 has recently received considerable attention due to its function in tumor progression and its decisive role in tumor resistance to chemotherapy. However, its role in pancreatic cancer has seldom been investigated. In this report, we provide evidence that RAD51, regulated by KRAS, promotes pancreatic cancer cell proliferation. Furthermore, RAD51 regulated aerobic glycolysis by targeting hypoxia inducible factor 1α (HIF1α).

**Methods:**

TCGA (The Cancer Genome Atlas) dataset analysis was used to examine the impact of RAD51 expression on overall survival of pancreatic cancer patients. Lentivirus-mediated transduction was used to silence RAD51 and KRAS expression. Quantitative real-time PCR and western blot analysis validated the efficacy of the knockdown effect. Analysis of the glycolysis process in pancreatic cancer cells was also performed. Cell proliferation was determined using a CCK-8 (Cell Counting Kit-8) proliferation assay.

**Results:**

Pancreatic cancer patients with higher levels of RAD51 exhibited worse survival. In pancreatic cancer cells, RAD51 positively regulated cell proliferation, decreased intracellular reactive oxygen species (ROS) production and increased the HIF1α protein level. KRAS/MEK/ERK activation increased RAD51 expression. In addition, RAD51 was a positive regulator of aerobic glycolysis.

**Conclusion:**

The present study reveals novel roles for RAD51 in pancreatic cancer that are associated with overall survival prediction, possibly through a mechanism involving regulation of aerobic glycolysis. These findings may provide new predictive and treatment targets for pancreatic cancer.

## Background

Pancreatic cancer is one of the most lethal cancers worldwide. Although significant progress has been made in diagnosis and treatment of the cancer, the overall survival of patients remains poor, with a 5-year overall survival rate of approximately 6% [1, 2]. Thus, there is an urgent need for a better understanding of the molecular mechanisms underlying pancreatic cancer.

It is well accepted that DNA damage is a recurring phenomenon in biology and plays an important role in cancer development [3, 4]. Human genomic DNA faces a large number of hazardous factors, including exogenous physical agents, spontaneous chemical reactions, and products of endogenous metabolism, such as reactive oxygen species (ROS), which can lead to DNA damage. To cope with and eliminate these unfavorable factors, cells have evolved the DNA damage response system to sense DNA damage, activate the cell cycle checkpoint and initiate the DNA repair process [5, 6]. Errors and defects in the DNA damage response lead to the accumulation of DNA lesions and induce instability, which eventually results in tumorigenesis [7]. In particular, germline mutations in some of the DNA repair machinery components have been discovered in pancreatic cancer, indicating the involvement of DNA damage response factors in pancreatic cancer development and progression [8, 9]. For example, approximately 10% of sporadic pancreatic adenocarcinoma patients harbor mutations of BRCA2, a tumor suppressor that plays important roles in DNA break repair and homologous recombination [10]. Germline heterozygous ATM (Ataxia Telangiectasia Mutated) mutations have been observed in families with hereditary pancreatic cancer, which further links the DNA damage machinery to pancreatic cancer [11, 12]. ATM and ATR (Ataxia Telangiectasia And Rad3 Related) can phosphorylate a number of substrates, including the checkpoint kinase CHK1 (Check point kinase 1), which mediates cell-cycle arrest to facilitate DNA repair [13]. Expression of CHK1 has been reported to be elevated in pancreatic cancer, and inhibitors targeting CHK1 have been shown to have clinical potential for treatment of pancreatic cancer [9, 14, 15].

As an important component of the DNA damage repair machinery, RAD51 has seldom been investigated in pancreatic cancer. In KRAS mutation-driven colorectal cancer, RAD51 has been reported to be transcribed by the KRAS downstream target MYC and to participate in the irradiation-induced DNA damage response and repair [16, 17]. However, the role of RAD51 in pancreatic cancer cell proliferation and other hallmarks has rarely been reported. In this study, we examined the correlation between RAD51 and KRAS mutation and the impact of RAD51 on cell proliferation and glucose metabolism reprogramming in pancreatic cancer. Furthermore, we attempted to identify the underlying molecular mechanism and found that RAD51 participates in ROS production and HIF1α protein level regulation. Collectively, our results provide novel predictive and treatment targets for pancreatic cancer.

## Materials and methods

### Cell culture

PANC-1 and MiaPaCa-2 cell lines were obtained from ATCC and cultured according to the standard protocols provided by ATCC. PANC-1 cells were cultured in Dulbecco’s modified Eagle’s medium (DMEM) containing fetal bovine serum (FBS) at a final concentration of 10%. MiaPaCa-2 cells were cultured in DMEM with 10% FBS and horse serum at a concentration of 2.5%. The cells were cultured in an incubator at 37 °C with 5% CO_2_.

### Western blotting analysis

Cells were harvested and lysed in RIPA buffer (20 mM Tris/HCl, pH 8.0, 150 mM NaCl, 20 μM EDTA, 1% NP40, 10% glycerol) supplemented with protease and phosphatase inhibitors for 10 min. Cell debris was removed by centrifugation at 12,000 rpm for 20 min at 4 °C. The concentrations of the lysates were determined using a BCA protein assay reagent kit (Pierce Biotechnology, Illinois, USA). Total protein lysate (20 μg) was subjected to electrophoresis in a denaturing 10% SDS–polyacrylamide gel and then transferred to a membrane, followed by blotting with specific antibodies. The antibody against RAD51 was purchased from Abcam. Antibodies targeting β-actin, KRAS and HIF1α were purchased from Proteintech (Illinois, USA).

### RNA isolation and quantitative real-time PCR

TRIzol reagent (Invitrogen, Massachusetts, USA) was utilized to extract total RNA. TaKaRa’s PrimeScript RT reagent kit was used for reverse transcription. The expression levels of the indicated genes were determined via quantitative real-time PCR using an ABI 7900HT Real-Time PCR system (Applied Biosystems, Massachusetts, USA). All reactions were run in triplicate. Primer sequences are listed in Table 1.

**Table 1:** Primer sequences used in the study

RAD51 forward	5′-GAGGTGAGCTTTCAGCCAGGCAGA-3′
RAD51 reverse	5′-TCTGGTTGTTGATGCATGGGCGA-3′
GLUT1 forward	5′-CTTTGTGGCCTTCTTTGAAGT-3′
GLUT1 reverse	5′-CCACACAGTTGCTCCACAT-3′
HK2 forward	5′-GATTGTCCGTAACATTCTCATCGA-3′
HK2 reverse	5′-TGTCTTGAGCCGCTCTGAGAT-3′
LDHA forward	5′-TGGAGATTCCAGTGTGCCTGTATGG-3′
LDHA reverse	5′-CACCTCATAAGCACTCTCAACCACC-3′
β-actin forward	5′-CTACGTCGCCCTGGACTTCGAGC-3′
β-actin reverse	5′-GATGGAGCCGCCGATCCACACGG-3′

### Lentivirus production and stable cell line selection

The pLKO.1 TRC cloning vector (Addgene plasmid: 10878) was used to express shRNA against RAD51. The 21-bp oligonucleotides targeting RAD51 were 5′-CGCCCTTTACAGAACAGACTA-3′ and 5′-TTAGAGCAGTGTGGCATAAAT-3′. Lentiviral particles were produced by co-transfection of HEK-293T cells with pLKO.1-shRAD51 constructs, psPAX2 and pMD2.G at a ratio of 4:3:1. The lentivirus was harvested 48 h after transfection. Stable cell lines were screened by infecting pancreatic cancer cells with lentiviral particles and subsequent puromycin selection.

### CCK-8 proliferation assay

Cell variability was assayed using a CCK-8 assay kit (Dojindo, Kumamoto, Japan), and the procedures were carried out according to the manufacturer’s protocol.

### Colony-formation capacity assessment

PANC-1 and MiaPaCa-2 cells (5 × 10^2^) stably expressing shRAD51 and the relative control cells were seeded in 6-well plates. The cells were cultured for 14 days, and 4% paraformaldehyde was used to fix the cells, followed by staining with 1% crystal violet. The colonies were subsequently counted.

### Glycolysis analysis

A Glucose uptake colorimetric assay kit (Biovision, California, USA) and Lactate colorimetric assay kit (Biovision, California, USA) were used to measure glucose utilization in pancreatic cancer cells.

### Measurement of oxygen consumption rate (OCR) and extracellular acidification rate (ECAR)

Cellular mitochondrial respiration and glycolytic capacity were measured using a Seahorse Bioscience XF96 Extracellular Flux Analyzer (Seahorse Bioscience, Massachusetts, USA). In brief, 4 × 10^4^ cells were seeded into 96-well plates and incubated overnight. After the cells were washed with Seahorse buffer (DMEM with phenol red containing 25 mM glucose, 2 mM sodium pyruvate, and 2 mM glutamine), 175 μl of Seahorse buffer plus 25 μl each of 1 μM oligomycin, 1 μM FCCP, and 1 μM rotenone were automatically injected to measure the OCR and 25 μl each of 10 mM glucose, 1 μM oligomycin, and 100 mM 2-deoxy-glucose (2-DG) were added to measure the ECAR. The OCR and ECAR values were calculated after normalization to cell number and are plotted as the mean ± SD [18].

### Detection of intracellular ROS

Intracellular ROS were detected using an oxidation-sensitive fluorescent probe (DCFH-DA) (Beyotime, Jiangsu, China). Cells were washed twice in phosphate-buffered saline (PBS). The samples were then incubated with 10 µmol/l DCFH-DA at 37 °C for 20 min according to the manufacturer’s instructions. DCFH-DA was deacetylated intracellularly by nonspecific esterases and further oxidized by ROS to the fluorescent compound 2,7-dichlorofluorescein (DCF). DCF fluorescence was detected using a FACScan flow cytometer.

### TCGA dataset analysis

TCGA-PAAD on RNA expression (Level 3) in pancreatic cancer patients in terms of RNA-seq with Expectation–Maximization was downloaded from the Cancer Genomics Browser of the University of California, Santa Cruz (UCSC) (https://genome-cancer.ucsc.edu/). In total, 160 primary pancreatic cancer samples from patients with detailed expression data were chosen from the updated TCGA database according to the parameters mentioned. Detailed demographics of these patients were characterized by the TCGA consortium.

### Statistical analyses

Statistical analyses were performed with SPSS software (version 17.0, IBM Corp., Armonk, NY, USA) using the independent Student’s *t*-test (two-tailed) or one-way analysis of variance (ANOVA). Logistic regression was used to determine correlations between RAD51, GLUT1, HK2 and LDHA expression levels and clinicopathological characteristics in the TCGA cohorts. A two-sided *P* value of < 0.05 was considered statistically significant.

## Results

### RAD51 expression predicted prognosis in pancreatic cancer

**Fig. 1:**
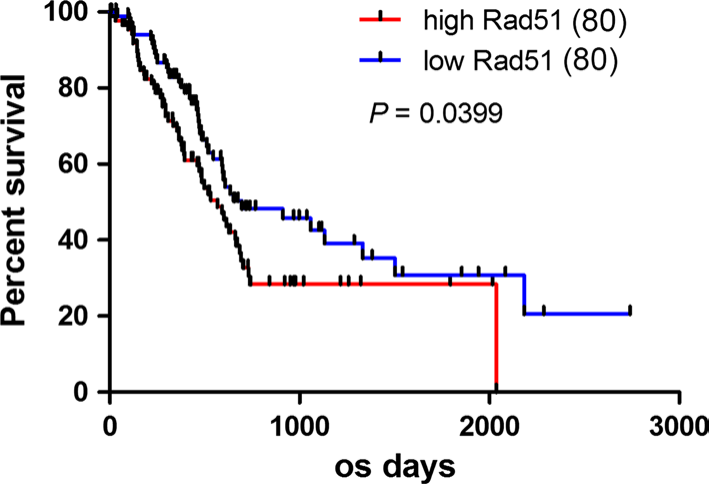
RAD51 expression predicts prognosis in pancreatic cancer. RAD51 expression in TCGA-included patients predicted prognosis, and patients with higher RAD51 exhibited a worse prognosis

To validate the role of RAD51 expression in pancreatic cancer prognosis, we used TCGA database to examine the correlation between RAD51 expression and overall survival of pancreatic cancer patients. The results demonstrated that patients with higher levels of RAD51 exhibited worse survival (Fig. 1a). The relationships between RAD51 expression and clinicopathological features are shown in Table 2.

**Table 2:** The role of RAD51 expression in clinicopathological features

Characteristics	No.	Rad51-Low	Rad51-High	*P* value
		80	80	
Age (years)				0.0915
< 60	37	14	23	
≥ 60	123	66	57	
Gender				0.5250
Female	72	34	38	
Male	88	46	42	
Tumor size (cm)				0.8732
< 4.0	91	45	46	
≥ 4.0	69	35	34	
Tumor differentiation				0.4902
Well/moderate	112	58	54	
Poor	48	22	26	
Pathological N				0.5978
N0	45	24	21	
N1	115	56	59	
Pathological M				0.1203
M0	156	76	80	
M1	4	4	0	
Pathological T				0.0960
T1/T2	28	18	10	
T3/T4	132	62	70	
Stage				0.5926
I–IIA	43	23	20	
IIB–IV	117	57	60	

### RAD51 promotes proliferation of pancreatic cancer cells

**Fig. 2:**
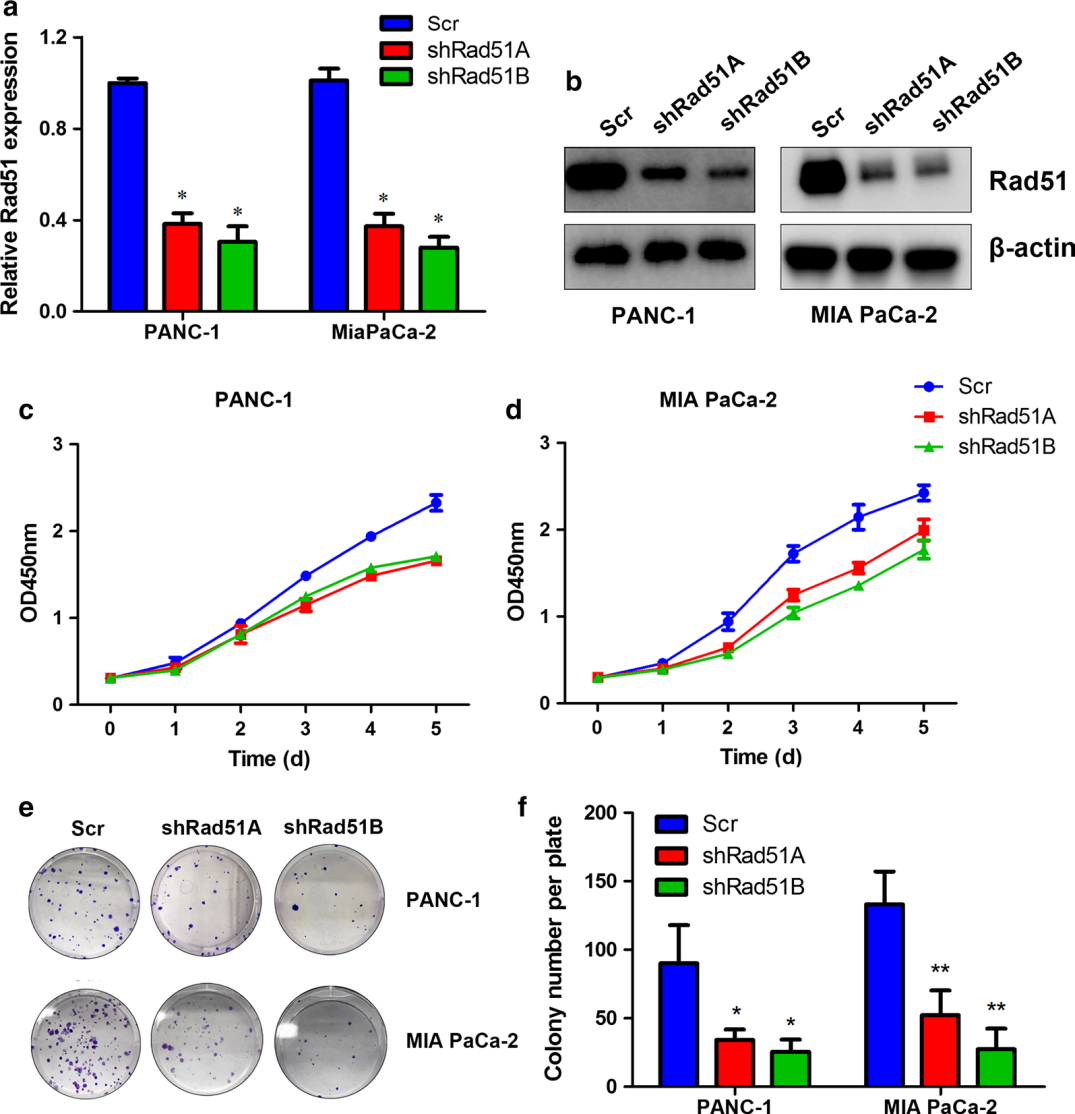
RAD51 regulates proliferation of pancreatic cancer cells. **a** Quantitative PCR results validated RAD51 knockdown efficiency. **b** The effect of RAD51 knockdown was further confirmed by western blot analysis. **c**, **d** CCK-8 proliferation assays demonstrated that RAD51 knockdown inhibited proliferation of PANC-1 and MiaPaCa-2 cells. **e**–**h** Colony formation assays demonstrated that silencing of RAD51 inhibited the clone formation capacity of PANC-1 and MiaPaCa-2 cells

To assess the impact of RAD51 on pancreatic cancer cell proliferation, we silenced RAD51 expression in PANC-1 and MIA PaCa-2 cells. Quantitative real-time PCR and western blot analysis validated the efficacy of the knockdown effect (Fig. 2a, b). Subsequently, we performed CCK8 proliferation assays to confirm the influence of RAD51 on cell viability. The CCK-8 results indicated that RAD51 knockdown significantly attenuated proliferation of PANC-1 and MiaPaCa-2 cells (Fig. 2c, d). Colony formation assays were also performed to further validate the role of RAD51 in pancreatic cancer cell proliferation. It was found that silencing RAD51 expression significantly attenuated the colony formation capacity of PANC-1 and MiaPaCa-2 cells (Fig. 2e, h). Collectively, these results suggest that RAD51 can positively regulate pancreatic cancer cell proliferation.

### RAD51 is regulated by oncogenic KRAS

**Fig. 3:**
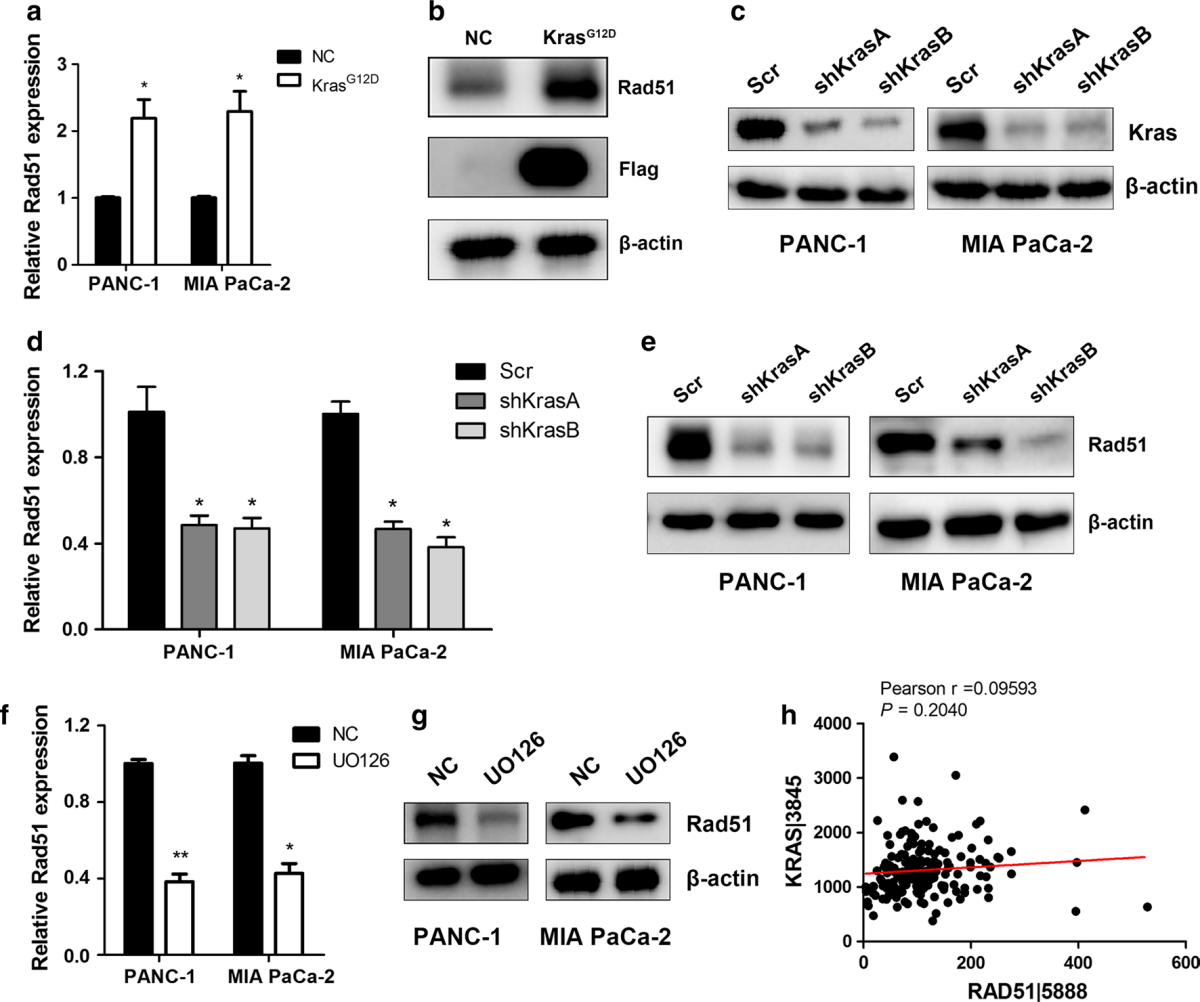
RAD51 is a downstream target of oncogenic KRAS. **a**, **b** KRAS G12D introduction into HEK293T cells increased the transcriptional and protein levels of RAD51. **c** KRAS expression was silenced in PANC-1 and MiaPaCa-2 cells. **d**, **e** KRAS knockdown decreased RAD51 mRNA and protein levels in PANC-1 and MiaPaCa-2 cells. **f**, **g** UO126 treatment decreased RAD51 expression in PANC-1 and MiaPaCa-2 cells. **h** RAD51 expression was positively and significantly correlated with KRAS expression in TCGA-included pancreatic cancer patients

KRAS mutation has been reported to be the driving force for pancreatic cancer oncogenesis and progression. We supposed that RAD51 might be a downstream target of KRAS. First, we overexpressed KRAS G12D in HEK293T cells, and upon KRAS G12D introduction, we observed increases in RAD51 mRNA and protein levels (Fig. 3a, b). We then silenced KRAS expression in KRAS mutated PANC-1 and MiaPaCa-2 cells (Fig. 3c). Subsequent quantitative PCR and western blot results demonstrated that KRAS knockdown resulted in decreased RAD51 transcription and protein levels (Fig. 3d, e). KRAS mutation resulted in constitutive activation of MEK/ERK. We inhibited this pathway using the MEK inhibitor UO126. Treatment with UO126 decreased RAD51 mRNA and protein expression levels (Fig. 3f, g). Furthermore, we analyzed the correlation between RAD51 expression and KRAS expression in TCGA-included patients, and we found that KRAS expression was positively correlated with RAD51 expression (Fig. 3h). Therefore, the present results indicate that KRAS/MEK/ERK activation can increase RAD51 expression in pancreatic cancer cells.

### RAD51 regulates ROS generation and HIF1α protein levels

**Fig. 4:**
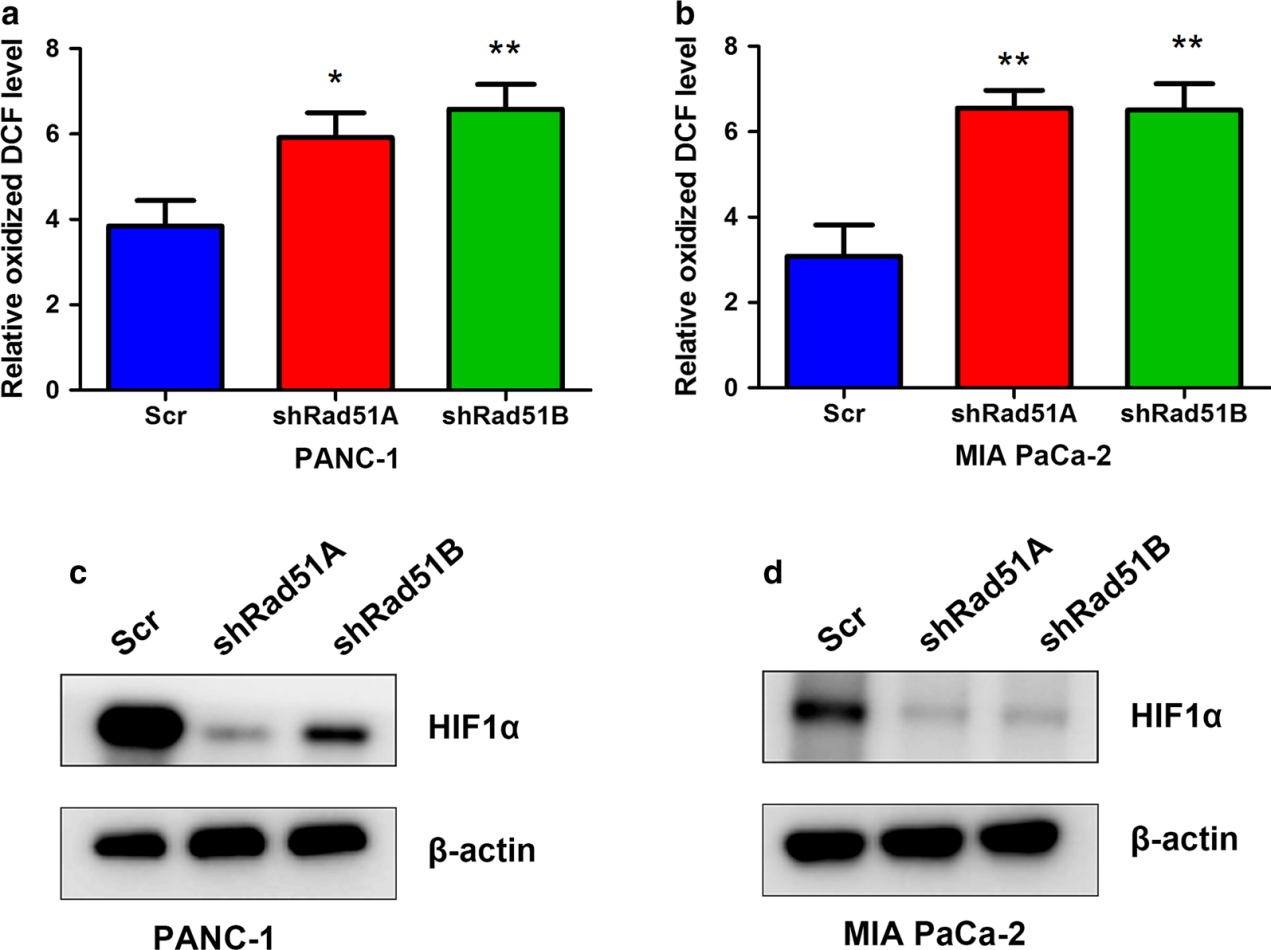
RAD51 regulates ROS production and HIF1α protein levels. **a**, **b** RAD51 knockdown decreased intracellular ROS levels in PANC-1 and MiaPaCa-2 cells. **c**, **d** Silencing of RAD51 in PANC-1 and MiaPaCa-2 cells decreased HIF1α protein levels in these cells

Previous studies have indicated that increased intracellular ROS levels can lead to activation of the DNA damage and repair pathway. However, the impact of the DNA damage and repair machinery on ROS production has seldom been studied. Thus, we assessed the impact of RAD51 silencing on ROS production in PANC-1 and MiaPaCa-2 cells. It was observed that silencing RAD51 significantly increased intracellular ROS production in pancreatic cancer cells (Fig. 4a, b). Because ROS accumulation in the cell can lead to changes in the stability of HIF1α, we assessed the impact of RAD51 on HIF1α. As expected, in RAD51-silenced PANC-1 and Mia PaCa-2 cells, we detected a significant decrease in HIF1α protein levels, supporting the positive impact of RAD51 on HIF1α protein level regulation (Fig. 4c, d).

### RAD51 regulated aerobic glycolysis in pancreatic cancer cells

**Fig. 5:**
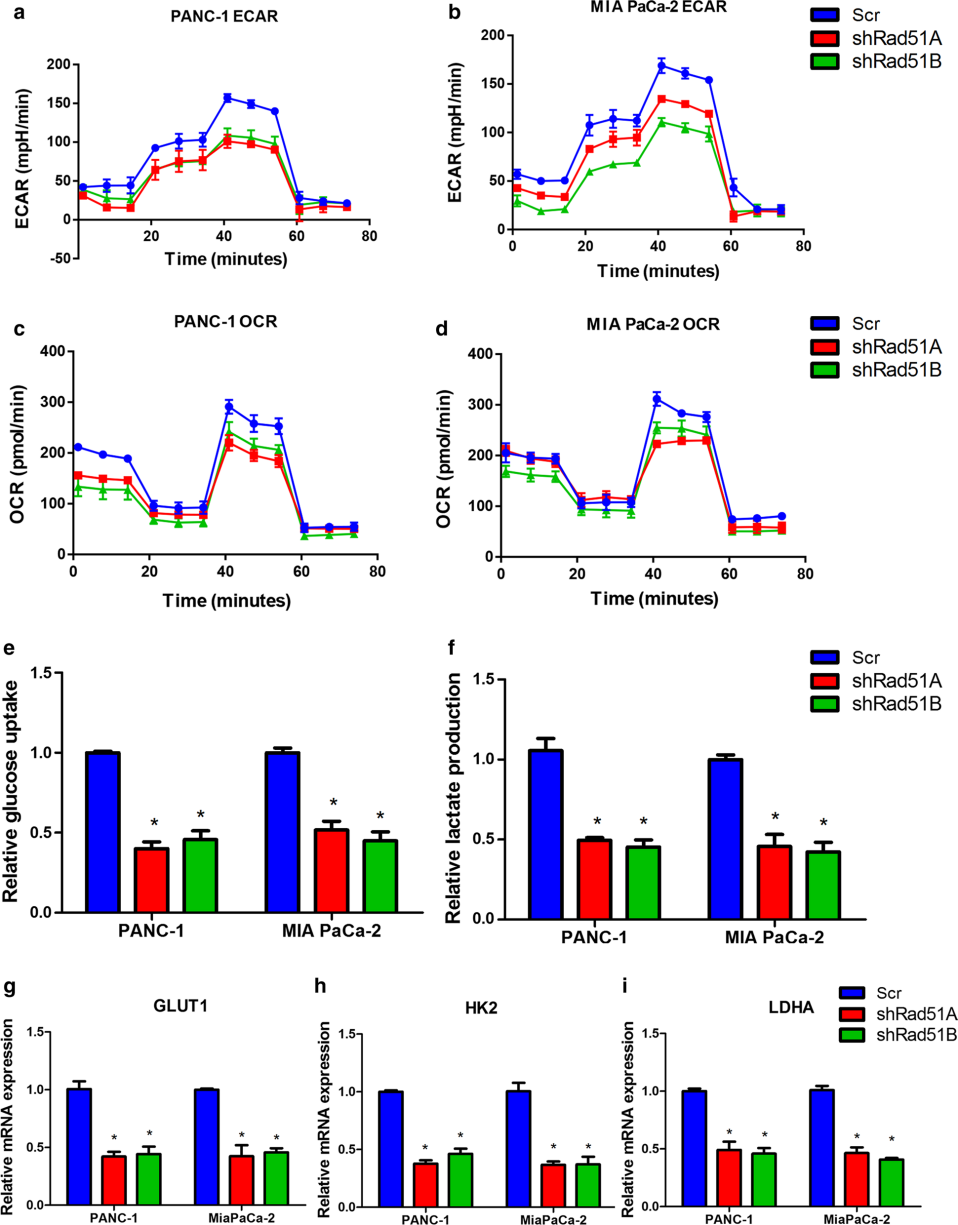
RAD51 regulates aerobic glycolysis in pancreatic cancer cells. **a**, **b** RAD51 knockdown inhibited aerobic glycolysis as demonstrated by ECAR measurements. **c**, **d** RAD51 knockdown increased OCR values in PANC-1 and MiaPaCa-2 cells, further demonstrating the negative role of RAD51 in mitochondrial respiration. **e**, **f** RAD51 knockdown decreased lactate production in PANC-1 and MiaPaCa-2 cells. **g**, **h** Silencing of RAD51 expression inhibited the expression of key glycolytic genes, including GLUT1, HK2 and LDHA, in PANC-1 and MiaPaCa-2 cells

As discussed above, RAD51 positively regulated HIF1α protein levels, and HIF1α is also a regulator of glucose metabolism transformation. Thus, we assessed the influence of RAD51 on glycolysis in pancreatic cancer cells. Knockdown of RAD51 inhibited glycolysis, which was reflected in the Seahorse ECAR measurement (Fig. 5a, b). During the glycolysis process, mitochondrial respiration was inhibited, leading to a decrease in the OCR. The OCR values in RAD51-silenced pancreatic cancer cells were then measured. The Seahorse extracellular flux analyzer results showed that decreased RAD51 expression increased the OCR value, further supporting the hypothesis that RAD51 is a positive regulator of aerobic glycolysis (Fig. 5c, d). Furthermore, we assessed the impact of RAD51 on lactate production, the product of glucose that is metabolized through glycolysis (Fig. 5e, f). Aerobic glycolysis is a process catalyzed by a series of glycolytic genes and involves the intake of glucose metabolized to lactate. Of these glycolytic genes, some are HIF1α targets, including GLUT1, HK2 and LDHA. We assessed the influence of RAD51 on the expression of these genes. As shown in Fig. 5g–i, RAD51 knockdown decreased the expression of GLUT1, HK2 and LDHA, further validating the contribution of RAD51 to glycolysis.

### RAD51 is positively correlated with glycolytic genes in pancreatic cancer patients

**Fig. 6:**
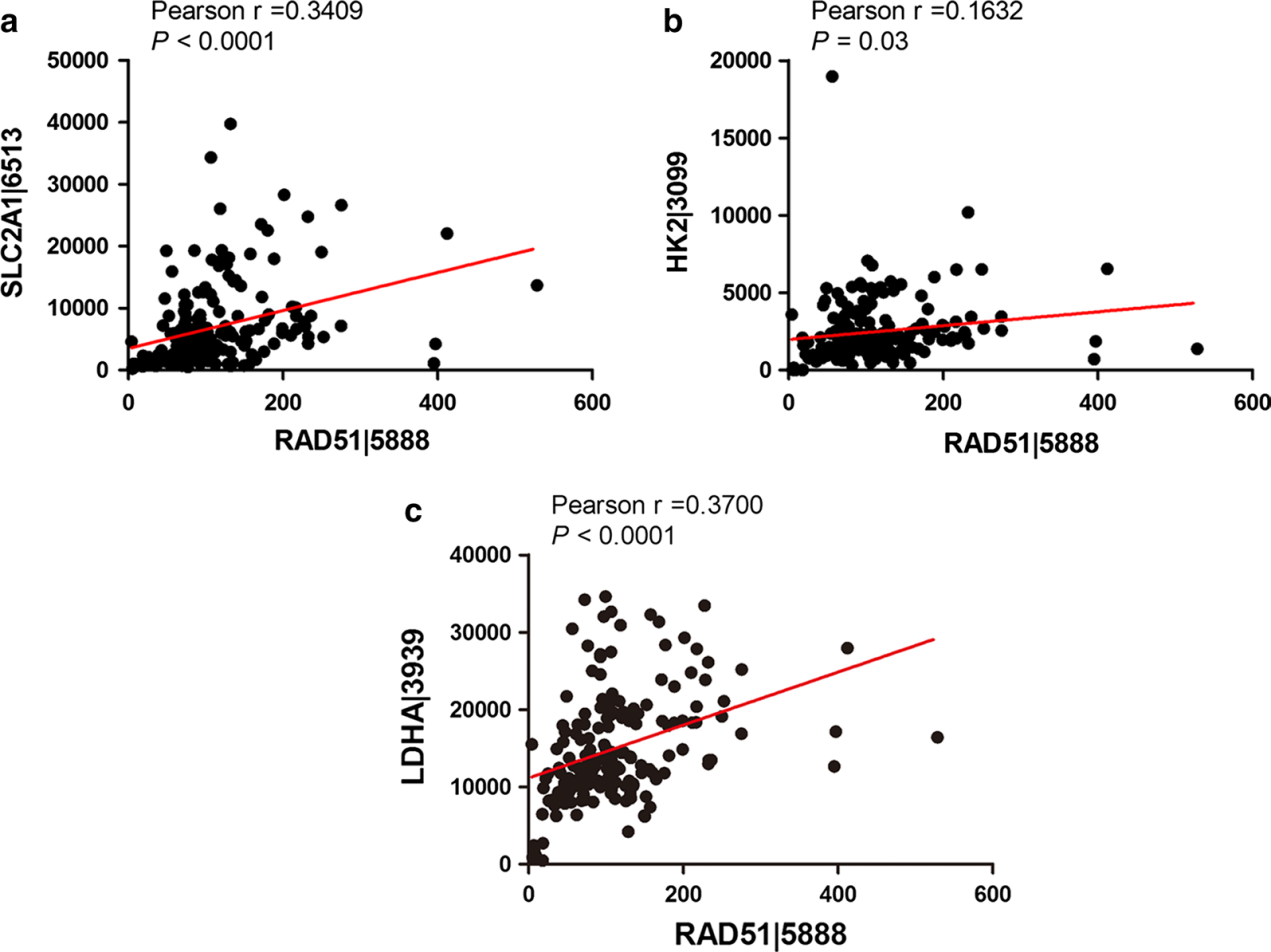
RAD51 was positively correlated with glycolytic gene expression levels in pancreatic cancer patients. **a**–**c** RAD51 was positively and significantly correlated with glycolytic genes, including GLUT1, HK2 and LDHA, in TCGA-included pancreatic cancer patients

To further confirm the above in vitro observations, we explored the correlation between RAD51 expression and HIF1α-targeted glycolytic enzymes in pancreatic cancer patients. Through expression analysis using the TCGA database, we found that RAD51 was positively and significantly correlated with GLUT1, HK2 and LDHA expression in TCGA-included patients, further validating the contribution of RAD51 to glycolysis (Fig. 6a–c).

## Discussion

Pancreatic cancer is one of the most lethal cancers and has an extremely poor overall survival rate. Although much progress has been made in diagnosis and treatment of this disease, the 5-year overall survival rate remains poor. Thus, there is an urgent need for a better understanding of the molecular mechanisms underlying the disease.

**Fig. 7:**
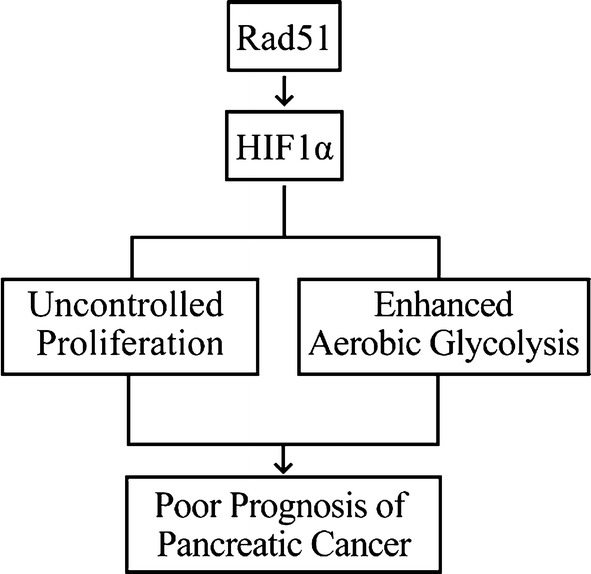
Schematic representation of a model illustrating the mechanism by which RAD51 promotes pancreatic cancer cell proliferation

The important contribution of aberrant metabolism to the formation and maintenance of malignancies has received much more attention in recent years, and cancer cell metabolism is considered one of the hallmarks of cancer [19–21]. RAD51 is a well-characterized factor in the DNA damage and repair process; however, its role in pancreatic cancer has scarcely been reported. In this report, we found that elevated RAD51 expression predicted worse prognosis in pancreatic cancer patients. Our mechanism studies indicated that RAD51 promoted pancreatic cancer progression by enhancing aerobic glycolysis via HIF1α (Fig. 7).

The clinical and biological functions of RAD51 expression in cancer are still under investigation. Moreover, how RAD51 expression is regulated in pancreatic cancer has seldom been reported. In the present study, our results demonstrated that introduction of KRAS G12D mutation, a mutation frequently observed in pancreatic cancer, could increase Rad51 mRNA and protein expression levels. When KRAS was silenced, RAD51 expression decreased in pancreatic cancer cells lines that harbor KRAS mutation. These results demonstrated that KRAS mutation or overexpression could increase RAD51 expression in pancreatic cancer cells. The decisive signaling cascade involved in the ability of KRAS mutation to drive malignancy is the MEK/ERK pathway. In pancreatic cancer cells, we inhibited the MEK/ERK axis using UO126. When pancreatic cancer cells were treated with UO126, we observed a decrease in RAD51 mRNA and protein levels. These results further confirm that the KRAS/MEK/ERK pathway might in part contribute to the expression of RAD51 in pancreatic cancer cells. Previous studies have shown the important functions of nuclear RAD51 expression in chemotherapy and radiotherapy resistance, which depend on its classical roles in DNA damage and repair [22–25]. Recently, the effect of aberrant cancer cell metabolism on diverse malignant behaviors has been demonstrated [26, 27]. Furthermore, cancer cell metabolism has been shown to contribute to regulation of chemotherapy and radiotherapy resistance [28–30]. Intrinsic and acquired chemotherapy and radiotherapy resistance is caused by aberrations in the DNA damage and repair system. Previous studies have demonstrated that genotoxic stress in cancer cells triggers a critical block in cancer cell metabolism, which is required for the DNA damage and repair response [31, 32]. Inspired by these observations, we examined the contribution of RAD51 to cancer cell metabolism, especially the glycolysis process. Our studies support the hypothesis that through the DNA damage and repair machinery RAD51 might contribute to cancer cell glycolysis by regulating the HIF1α transcriptional process. Although we observed that silencing RAD51 inhibited HIF1α at the protein level, we did not investigate how RAD51 regulates HIF1α at the protein level. The HIF1α protein level is tightly controlled by post-translational modifications, including acetylation, hydroxylation and ubiquitination [33, 34]. RAD51 may interact and regulate the protein stability of HIF1α, especially under genotoxic conditions, leading to enhanced glycolysis and contributing to chemotherapy and radiotherapy resistance [35]. HIF1α protein stabilization and upregulation is regarded as an important factor in chemotherapy and radiotherapy resistance in pancreatic cancer [35, 36]. The present study may shed light on approaches to prevent chemotherapy and radiotherapy resistance in pancreatic cancer by targeting the RAD51/HIF1α axis.

Glycolysis has been reported to support cancer cells by providing building materials for macromolecule synthesis and ATP production [37, 38]. Furthermore, through glycolysis, cancer cells can create an acidic microenvironment via lactate accumulation, resulting in extracellular matrix destruction that favors metastasis [39]. In the present study, RAD51 was demonstrated to have a role in regulation of glycolysis; however, its role in metastasis has not been sufficiently studied. Metastasis has been shown to be an important factor in cancer-related deaths in patients with pancreatic cancer [40]. Thus, increased RAD51 expression in pancreatic cancer might regulate metastasis. RAD51 has been reported to maintain metastasis in triple-negative breast cancer, and RAD51 inhibition sensitizes breast cancer stem cells to PARP inhibitor [41]. Metastasis has been implicated as one of the factors contributing to chemotherapy and radiotherapy resistance. For example, the epithelial–mesenchymal transition (EMT) process not only contributes to metastasis but also confers cancer cell resistance to chemotherapy and radiotherapy [42]. Thus, uncovering the role of RAD51 in metastasis of pancreatic cancer and elucidating the underlying mechanism might lead to inhibition of metastasis and reversal of chemotherapy and radiotherapy resistance in pancreatic cancer.

Collectively, our findings identified novel roles for RAD51 in pancreatic cancer in relation to prediction of overall survival, as well as the possible underlying mechanisms. The results will provide new predictive and treatment targets for pancreatic cancer and a new direction for uncovering the DNA damage and repair machinery in pancreatic cancer oncogenesis and progression.

## Conclusion

Our present study revealed that Rad51 is a predictive marker for overall survival in pancreatic cancer. Mechanism studies demonstrated that Rad51 is a downstream target of Kras. Moreover, Rad51 positively regulates aerobic glycolysis in pancreatic cancer cells by regulating HIF1α protein stability and the HIF1α-targeted transcriptional program.

## Data Availability

All data analyzed and displayed in the present manuscript are available from the corresponding author upon reasonable request.

## Abbreviations

TCGA: The Cancer Genome Atlas
HIF1α: hypoxia inducible factor 1α
ROS: reactive oxygen species
GLUT1: glucose transporter 1
HK2: hexokinase 2
LDHA: lactate dehydrogenase A
